# Preoperative Administration of Levosimendan to Prevent Low Cardiac Output Syndrome Following Pediatric Cardiac Surgery: A Retrospective Study

**DOI:** 10.3390/clinpract16030063

**Published:** 2026-03-22

**Authors:** Laurence Boillat, Laure Pache-Wannaz, Guillaume Maitre, Frida Rizzati, Maria Pérez Marin, Vivianne Chanez, Stefano Di Bernardo, Maria-Helena Perez

**Affiliations:** 1 Pediatric Intensive Care Unit, Women-Mother-Child Department, Lausanne University Hospital, 1011 Lausanne, Switzerland; guillaume.maitre@chuv.ch (G.M.); frida.rizzati@chuv.ch (F.R.); maria.perez-marin@chuv.ch (M.P.M.); vivianne.chanez@chuv.ch (V.C.); marie-helene.perez@chuv.ch (M.-H.P.); 2 Pediatric Cardiology, Women-Mother-Child Department, Lausanne University Hospital, 1011 Lausanne, Switzerland; laure.pache-wannaz@chuv.ch (L.P.-W.); stefano.di-bernardo@chuv.ch (S.D.B.)

**Keywords:** levosimendan, low cardiac output syndrome, congenital heart disease

## Abstract

**Background:** Low cardiac output syndrome (LCOS) is a significant cause of postoperative morbidity and mortality in children with congenital heart disease. Prophylactic levosimendan is increasingly used to prevent LCOS, but its superiority to other strategies remains unproven. Based on the pharmacokinetics of levosimendan, we hypothesize that preoperative administration is beneficial for preventing LCOS in a specifically at-risk population. **Methods**: This is a retrospective single-center cohort study in a tertiary pediatric intensive care unit. All patients under one year of age undergoing surgery for congenital heart disease using cardiopulmonary bypass and receiving levosimendan within 24 h before or after surgery were included and classified into two groups: preoperative and postoperative administration. **Results**: Overall, 107 patients were included. Fifty-three patients (49.5%) received levosimendan before surgery, with significantly lower mortality, fewer LCOS markers, and lower LCOS scores compared to patients receiving levosimendan after surgery. Although not significant, the use of extracorporeal membrane oxygenation, renal replacement therapy, and temperature control was also lower in the preoperative group. There was no difference in mechanical ventilation duration and length of stay. **Conclusions**: Preoperative administration of levosimendan seems associated with a lower incidence of LCOS and reduced mortality in high-risk children with congenital heart surgery.

## 1. Introduction

Children with congenital heart disease undergoing cardiac surgery are at high risk of developing low cardiac output syndrome (LCOS), which imposes a significant burden of postoperative morbidity and mortality. LCOS arises from an imbalance between oxygen demand and supply and is multifactorial, affecting up to 25% of children undergoing heart surgery [1]. The combination of pre-existing cardiac conditions and the consequences of open-heart surgery contributes to the development of LCOS. Inherent patient risk factors include young age, particularly during the neonatal period, ventricular myocardial hypertrophy, chronic hypoxemia, or myocardial dysfunction. Additionally, several factors emerge during surgery that may contribute to LCOS, including ischemia secondary to aortic cross-clamping, residual effects of cardioplegia, myocardial reperfusion injury, activation of inflammatory cascades and the complement system, and alterations in pulmonary and systemic vascular resistances [1,2]. LCOS prolongs mechanical ventilation and hospital stay, and increases the risk of cardiopulmonary arrest, thereby increasing the need for cardiopulmonary resuscitation or extracorporeal circulation support [3,4,5]. It is also recognized as the leading cause of death in children with congenital heart disease [6].

Strategies to prevent LCOS in the perioperative management of these children are necessary. The cornerstones of LCOS treatment include catecholamines, phosphodiesterase inhibitors, and calcium sensitizers. However, the lack of evidence favoring one treatment over another has led to the absence of evidence-based guidelines and a notable disparity in management practices across pediatric intensive care units (PICUs) [7,8,9].

Levosimendan is a calcium-sensitizing inotropic agent that enhances myocardial contractility without raising intracellular calcium levels or myocardial oxygen consumption, thereby limiting pro-arrhythmic and ischemic effects while also exerting vasodilatory and cardioprotective actions through the opening of ATP-sensitive K^+^ channels [10,11,12,13,14]. Administered as a continuous infusion over 24–48 h, it is metabolized to OR-1896, an active metabolite with a long half-life, that accounts for sustained hemodynamic effects lasting up to 7–14 days after treatment discontinuation [13,15,16,17].

Levosimendan has demonstrated effectiveness, safety, and good tolerability in children [15]. Recent studies have shown that its use in pediatric postoperative congenital heart surgery slightly reduces the incidence of LCOS and may also reduce mortality compared with placebo [18,19,20]. So far, studies have failed to demonstrate its superiority over standard inotropic therapy, especially milrinone, which has been widely used to prevent LCOS since the PRIMACORP study in 2003 [4,21]. Nevertheless, the safety and tolerability of levosimendan in pediatrics provide a reassuring foundation for its potential in pediatric cardiac care.

Our local use of levosimendan has evolved. Initially administered as a rescue therapy [22], it has since become the postoperative treatment of choice for patients at risk of developing LCOS or showing signs of decreased postoperative cardiac output. Since 2010, levosimendan has been increasingly administered preoperatively to selected patients considered at risk, yielding encouraging results [23]. Based on its unique pharmacokinetic properties, we hypothesize that preoperative administration is beneficial for preventing LCOS. This retrospective study aims to analyze this preventive strategy for LCOS by comparing LCOS incidence among newborns and infants undergoing open-heart surgery who received levosimendan preoperatively or postoperatively.

## 2. Materials and Methods

We conducted an observational, retrospective, single-center cohort study in the pediatric intensive care unit (PICU) at Lausanne University Hospital Center in Switzerland. This tertiary care teaching center performs approximately 200 pediatric open-heart surgeries annually. Our local ethics committee approved this study and waived informed consent.

We included all patients under one year of age who underwent corrective or palliative surgery for congenital heart disease with cardiopulmonary bypass (CPB) between 2005 and 2019 and received levosimendan within 24 h before or after surgery. Exclusion criteria were a Risk Adjustment for Congenital Heart Surgery (RACHS-1) score ≤ 2, a validated risk adjustment classification that stratifies congenital heart surgery procedures according to operative mortality risk, heart transplantation, and ventricular assist device implantation. Patients who required circulatory support with extracorporeal membrane oxygenation (ECMO) after surgery were included in the population descriptions; however, their LCOS variables were not analyzed.

Classification into preoperative or postoperative groups was based on the timing of levosimendan infusion initiation. The case group is the preoperative group (PRE), with levosimendan infusion initiated the day before surgery, usually 12 h before CPB initiation, and continued during and after surgery for a total of 48 h. The control group is the postoperative group (POST), receiving a 48 h course of levosimendan, starting within 24 h after the end of CPB. No selection or matching procedure was performed to obtain balanced group sizes.

The levosimendan administration protocol was identical for both groups. The continuous dose was 0.1 mcg/kg/min for 48 h, without a loading dose.

Data were retrospectively collected from the institutional computerized patient record (Soarian^®^ Siemens) and the patient data management system (Metavision^®^ iMDSoft) used in our PICU. The analyzed data included diagnosis, demographics, surgical and anesthetic procedures, hemodynamic and biological parameters, drug administration, renal replacement therapy (RRT), durations of invasive and non-invasive ventilation, delayed sternal closure, ECMO, length of stay, and mortality during the PICU stay. Metavision^®^ records patients’ hemodynamic data (heart rate, blood pressure, central venous pressure) every minute, hourly diuresis, medication administration, and laboratory results. Pediatric cardiologists performed echocardiograms, and data were collected from the Philips Xcelera and IntelliSpace Cardiovascular^®^ ultrasonography program.

The evaluation of cardiac output was based on monitoring multiple clinical parameters indicating decreased in cardiac output, including tachycardia, hypotension, increased lactatemia, decreased venous oxygen saturation (S_v_O_2_), increased difference between arterial and central venous oxygen saturation (D_av_SO_2_ = S_a_O_2_ − S_v_O_2_), increased difference between arterial and venous partial pressure of carbon dioxide (pCO_2_) (D_av_CO_2_ = P_v_CO_2_ − P_a_CO_2_), oliguria, and increased vasoactive–inotropic score (VIS) [4,24]. VIS was calculated as follows: dopamine (mcg/kg/min) + dobutamine (mcg/kg/min) + [100 × epinephrine (mcg/kg/min)] + [10 × milrinone (mcg/kg/min)] + [10,000 × vasopressin (U/kg/min)] + [100 × norepinephrine (mcg/kg/min)] [25]. To assess the incidence of LCOS, we created a composite score based on five criteria (Table 1). LCOS was defined as a score ≥ 3 points. These parameters were analyzed at 6 h intervals over 48 h. T0 corresponds to the patient’s arrival at the PICU after surgery.

Statistical analysis was performed using Stata 18.0 (StatCorp). Discrete data are presented as absolute numbers and (percentages) and analyzed using Pearson’s Chi-square test. Continuous data are presented as median and [interquartile range] and analyzed using the Wilcoxon–Mann–Whitney test. Statistical significance was inferred at a value of *p* < 0.05.

## 3. Results

A total of 107 patients were included, with 53 (49.5%) in the case group (PRE) and 54 (50.5%) in the control group (POST). As shown in Table 2, the study groups did not differ in terms of gender, age, height, body surface area, cardiac diagnosis, RACHS-1 score, minimum body temperature, duration of CPB, or aortic cross-clamping. Only weight was significantly different at the time of surgery, with the PRE group having a lower median weight. Newborns (≤28 days old) represented 71% of our study population. Every patient received a 48 h course of levosimendan without a loading dose. No patients were treated preoperatively before 2009, and most patients (70%) in the PRE group underwent surgery after 2013.

**Figure 1:**
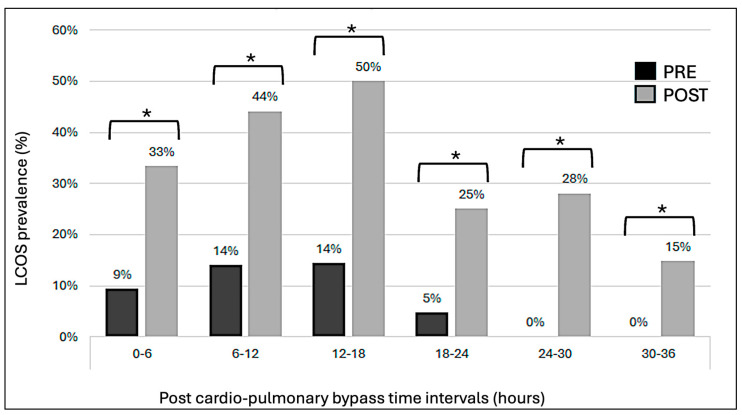
LCOS prevalence over time. Bars indicate the proportion of patients who developed LCOS according to our LCOS score. Significance is inferred with a *p*-value < 0.05 and identified with a *.

**Figure 2:**
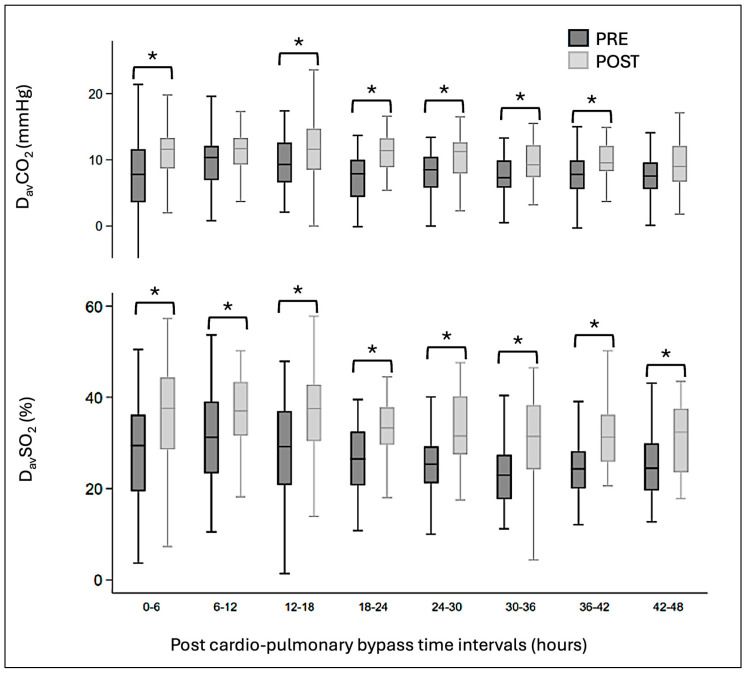
D_av_CO_2_ (difference between arterial and central venous pCO_2_) and D_av_SO_2_ (difference between arterial and central venous oxygen saturation) over time. Mean value, interquartile range, and minimal and maximal values are represented in the graph. Significance is inferred with a *p*-value < 0.05 and identified with a *.

**Figure 3:**
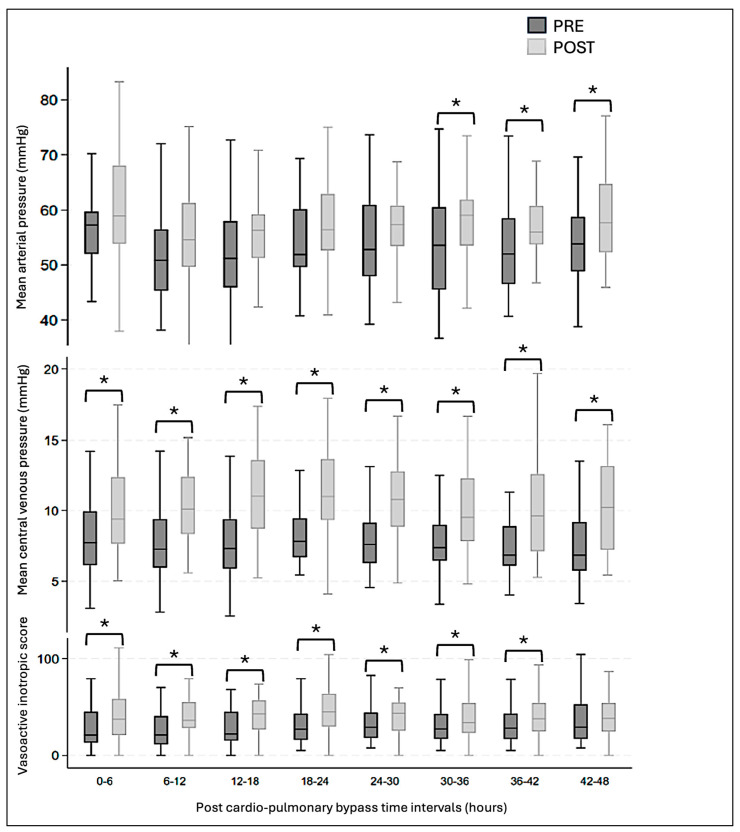
Mean arterial pressure, mean central venous pressure, and vasoactive inotropic score at 6 h intervals after cardio-pulmonary bypass. Mean value, interquartile range, and minimal and maximal values are represented in the graph. Significance is inferred with a *p*-value < 0.05 and identified with a *.

The preoperative administration of levosimendan was associated with a significantly lower LCOS score during the first 36 h after CPB (Figure 1). D_av_SO_2_ and D_av_CO_2_ were significantly lower in the PRE group (Figure 2). Mean arterial pressures remained similar in both groups during the first 30 h post-CBP and were then lower in the PRE group, without an increase in other LCOS markers. Mean venous pressures were significantly lower during the first 48 h post-operatively in the PRE group, resulting in perfusion pressures that were identical for both groups. The vasoactive inotropic score was significantly lower in the PRE group during the first 42 h after surgery (Figure 3). There was no difference in lactatemia or urine output. Mortality was significantly lower in the PRE group (6%) than in the POST group (19%) (*p* = 0.045).

Results for clinical outcome variables are presented in Table 3. Although not significant, the use of ECMO, RRT, and temperature control was lower in the PRE group than in the POST group. The duration of invasive mechanical ventilation was similar in both groups, with a median of 191 h (*p* = 0.52). Noninvasive ventilation was used more frequently in the PRE group, but total ventilation duration did not differ between the two groups. There was no difference in the length of PICU stay.

## 4. Discussion

Among the first studies to directly compare different timing strategies for levosimendan administration in infants and newborns undergoing cardiac surgery, our study shows that preoperative administration of levosimendan is associated with a significant reduction in the occurrence and severity of LCOS. Mortality was also significantly lower compared with postoperative administration. Several physiological markers, including lower D_av_CO_2_ and D_av_SO_2_, lower central venous pressure, reduced vasoactive–inotropic score (VIS), and shorter duration of active cooling system use, consistently suggest improved postoperative cardiac output in the PRE group.

Although the use of ECMO and RRT was numerically lower in the PRE group, these differences did not reach statistical significance and should therefore be interpreted with caution. Nevertheless, the overall trend may support the hypothesis of improved postoperative cardiovascular stability.

Non-invasive ventilation was used more frequently in the PRE group; however, the duration of non-invasive ventilation, as well as the total duration of ventilatory support, were similar between groups. This finding likely reflects evolving clinical practices, with more systematic implementation of post-extubation non-invasive ventilation over time rather than a direct effect of levosimendan administration.

Taken together, these findings suggest that preoperative levosimendan administration may contribute to improved perioperative cardiovascular adaptation, resulting in more stable postoperative hemodynamics and a reduced severity of LCOS rather than solely affecting isolated clinical endpoints.

Our use of levosimendan has evolved over the years, and the number of patients treated preoperatively has increased. These results corroborate the clinical observations in our PICU that followed this evolution. The timing of levosimendan administration is a critical factor in reducing the incidence of LCOS in our patients.

Based on levosimendan’s pharmacokinetics, preoperative administration allows therapeutic plasma levels to be achieved before surgery. Thus, the cardioprotective result is effective from the onset of myocardial injury, and the inotropic effect is evident after CPB removal. This timing of administration, known as “preconditioning,” has been studied in adults undergoing cardiac surgery.

Eris et al. showed that elective preoperative administration of levosimendan 12 h before coronary artery bypass graft (CABG) surgery in a population with preexisting severe left ventricular dysfunction (left ventricular ejection fraction (LVEF) < 30%) was associated with higher cardiac output, lower mortality, lower use of other inotropic or vasopressor drugs, less need for intra-aortic balloon pump (IABP) support and shorter ICU length of stay compared with peri or postoperative administration [26].

Levin et al. reported similar results in patients with severe left ventricular dysfunction (LVEF < 25%) who received levosimendan 24 h before surgery, compared with placebo [27]. Mortality, postoperative LCOS incidence, requirements for inotropic and vasopressor agents, and the need for IABP were significantly reduced. They concluded that patients at risk of developing postoperative left ventricular systolic dysfunction could benefit from early hemodynamic optimization, using levosimendan as a preconditioning agent. A recent meta-analysis also supports these conclusions [28]. Jimenez-Rivera et al. reported similar findings. They concluded that such a prevention strategy is beneficial not only for clinical outcomes but also for cost-effectiveness [29].

Schiefenhövel et al. analyzed the timing of levosimendan administration in patients with left ventricular systolic dysfunction undergoing on-pump cardiac surgery, comparing three administration times: the day before surgery, during surgery, or after surgery. They demonstrated that preoperative levosimendan (administered the day before surgery) in patients with left ventricular systolic dysfunction reduces mortality, length of stay, duration of mechanical ventilation, and the need for RRT compared with perioperative or postoperative administration [30].

Only a few pediatric studies have reported preoperative administration [10,31]. Abril-Molina et al. observed a non-significant decrease in cardiac enzymes, reflecting myocardial injury, and a significant reduction in lactatemia, along with improved oxygen delivery, in patients receiving levosimendan 12 h before cardiac surgery compared with those receiving placebo [31]. Although their dosing regimen (0.2 µg/kg/min for 36 h) differed from ours (0.1 µg/kg/min for 48 h), these results are largely in line with our findings, which also show improved postoperative hemodynamic parameters and a reduction in the severity of LCOS with preoperative levosimendan administration. Most pediatric randomized controlled trials (RCTs) have investigated postoperative levosimendan compared with other inotropic regimens and have demonstrated a reduction in LCOS but failed to show superiority for other outcomes, such as mortality, length of stay, or duration of ventilation [18,19,21,32]. We hypothesize that this lack of effect on these outcomes may be due to inadequate patient selection. Indeed, most RCTs studied patients with low RACHS-1 scores, who typically have a low incidence of postoperative LCOS, and excluded univentricular patients, newborns, or patients with preoperative hemodynamic instability or preexisting cardiac dysfunction, who are most at risk of developing LCOS after surgery. This hypothesis that patients “were not sick enough” to prove the effectiveness of levosimendan was addressed by Beretta-Piccoli et al. in their critique of Wang’s article [33,34], which studied patients with a low RACHS-1 score and normal preoperative cardiac function.

At our center, the preoperative administration of levosimendan is decided on a multidisciplinary basis before the intervention, considering patient-specific risk factors and the characteristics of their surgical procedures (estimated cardiopulmonary bypass time ≥ 180 min, estimated aortic cross-clamping time ≥ 90 min, preoperative systolic dysfunction, severe left or right ventricular hypertrophy with diastolic dysfunction, newborns and infants). Patients without significant risk factors do not receive prophylactic perfusion. To homogenize the population studied, we targeted patients under one year old with a RACHS-1 score ≥ 3, who were considered at risk of developing post-operative LCOS. Our results showed high mortality (19%) and an LCOS incidence of up to 50% in the POST group, which is higher than described in previous studies [18,21,35]. This highlights that our study population was adequately targeted to those most at risk of developing LCOS.

Most patients in the POST group developed signs of decreased cardiac output postoperatively and subsequently received levosimendan, which may explain why mortality was so high. The same observation was made in the adult population, which showed a higher mortality rate in patients receiving levosimendan after the installation of LCOS compared with its prophylactic administration [30].

Although well-defined and easily measurable in adults, cardiac output in children remains a challenging state to assess. Invasive measurement of the cardiac index is rarely performed in children, especially newborns and infants, due to technical and physiological limitations [36]. The lack of a validated LCOS definition for pediatric patients results in significant disparities in LCOS diagnosis. Most studies rely on associations among several variables or on subjective clinical assessment, leading to significant heterogeneity in RCT results [21].

Given the retrospective design and non-randomized treatment allocation, residual confounding cannot be excluded. Patients in the PRE group had a significantly lower median body weight at the time of surgery. Although statistically significant, this difference was not associated with outcome patterns, suggesting a clinically meaningful influence of body weight alone. Despite the absence of other significant demographic or clinical differences between the two groups, the study population shows heterogeneity in cardiac diagnoses and surgical procedures. The long study period represents an additional limitation of this study. Over the 15-year inclusion period, surgical techniques, perioperative management, and intensive care practices inevitably evolved and may have influenced patient outcomes independently of levosimendan administration. Importantly, the implementation of levosimendan in our institution occurred progressively rather than as a discrete change in practice, with a prolonged period during which postoperative and preoperative administration strategies overlapped. Consequently, treatment exposure was not strictly associated with a specific temporal era, making era-based stratification difficult to interpret and potentially introducing additional classification bias. Nevertheless, residual confounding related to temporal improvements in care cannot be completely excluded and should be considered when interpreting our findings.

In addition, because prophylactic administration is used, patients undergoing emergency surgery are automatically excluded from the PRE group, whereas this is not the case for the POST group. The small number of patients included in the study is also a limiting factor. Furthermore, patients in the PRE group were selected based on their risk of developing LCOS. In contrast, patients in the POST group were primarily those who already presented with signs of LCOS within the first few hours after surgery, which limits the interpretation of the results. The timing of levosimendan administration in the POST group was recorded as within 24 h after cardiopulmonary bypass. The exact start time could not be determined retrospectively, which may have introduced variability in the onset and progression of LCOS and represents a limitation of our study. Although preoperative administration is associated with better outcomes, we cannot definitively state that it reduces mortality compared with postoperative administration.

## 5. Conclusions

Preventing and treating low cardiac output syndrome in children with congenital heart disease remains a major focus of pediatric cardiac intensive care research. Our findings suggest that levosimendan may provide effective prophylaxis when administered preoperatively to selected high-risk newborns and infants. Prospective multicenter randomized controlled trials are warranted to confirm these observations.

## Figures and Tables

**Table 1:** Composite LCOS Score.

	0 Point	1 Point
VIS	≤25	>25
Urine output	≥0.5 mL/kg/h	<0.5 mL/kg/h
Arterial lactatemia	≤3 mmol/L	>3 mmol/L
D_av_CO_2_	≤10 mmHg	>10 mmHg
D_av_SO_2_	≤40%	>40%

LCOS was defined as a score ≥ 3 points. *VIS, vasoactive–inotropic score; DavCO2, difference between arterial and venous partial pressure of carbon dioxide; DavSO2, difference between arterial and central venous oxygen saturation.*

**Table 2:** Demographic and clinical characteristics.

	PRE Group (N = 53)	POST Group (N = 54)	*p*-Value
**Demographic data**			
Height (cm)	50 [49–52]	52 [49–58]	0.249
Weight (kg)	3.2 [2.9–3.6]	3.5 [3.1–4.4]	0.037 *
Body surface area (m^2^)	0.21 [0.20–0.23]	0.22 [0.20–0.25]	0.263
Age (days)	8 [6–22]	11 [7–74]	0.185
Gender male	26 (49)	33 (61)	0.210
Newborns (<30 days old)	41 (77)	35 (65)	0.15
**Cardiac diagnosis**			0.258
Aortic arch interruption or coarctation	9 (17)	7 (13)	
RVOTO	9 (17)	14 (26)	
Single ventricle	11 (21)	4 (7)	
TGA	18 (34)	20 (37)	
Others	6 (11)	9 (17)	
RACHS-1 score			0.433
RACHS 3	9 (17)	13 (27)	
RACHS 4	32 (62)	29 (59)	
RACHS 6	11 (21)	7 (14)	
**Perioperative data**			
Cardiopulmonary bypass time (min)	191 [156–240]	172 [146–225]	0.271
Aortic-cross-clamp time (min)	108 [76–130]	94 [74–114]	0.257
Minimal body temperature (°C)	31.5 [23.5–33.2]	30 [25–32.9]	0.95

Discrete data are expressed as absolute numbers (percentages), and continuous data are expressed as median [interquartile range]. Significance was determined with a *p*-value < 0.05, and significant differences are indicated with *. *RVOTO, right ventricular outflow tract obstruction; TGA, transposition of the great arteries; RACHS-1 score, Risk Adjustment for Congenital Heart Surgery (RACHS-1) score.*

**Table 3:** Postoperative data.

	**PRE Group (N = 53)**	**POST Group (N = 54)**	***p*-Value**
Mortality	3 (6)	10 (19)	0.045 *
ECMO	7 (13)	12 (22)	0.222
ECMO duration (days)	5.4 [4.4–10.9]	5.2 [4.1–6.8]	0.902
Renal replacement therapy	0 (0)	3 (6)	0.082
Pacemaker use	47 (89)	52 (96)	0.134
Cooling system	11 (21)	14 (26)	0.527
Cooling system (days)	0.1 [0.1–0.8]	0.6 [0.4–2.2]	0.032 *
Open chest	37 (70)	32 (59)	0.254
Open chest duration (days)	4.1 [3.0–6.5]	5.8 [3.0–7.8]	0.328
Invasive MV	52 (98)	53 (98)	0.989
Invasive MV (hours)	191.4 [104.4–306.8]	191.3 [138.0–360.8]	0.52
Noninvasive MV	41 (77)	30 (56)	0.017 *
Noninvasive MV (hours)	22 [17–35]	23 [13–46]	0.993
Total ventilation duration (hours)	208 [120–327]	195 [139–374]	0.667
Length of PICU stays (days)	19 [13–39.8]	15 [11–29]	0.247

Discrete data are expressed as absolute numbers (percentages), and continuous data are expressed as median [interquartile range]. Significance was determined with a *p*-value < 0.05, and significant differences are indicated with *. *ECMO, extracorporeal membrane oxygenation; MV, mechanical ventilation.*

## Data Availability

The data presented in this study are available upon request from the corresponding author due to ethical restrictions.

## Abbreviations

The following abbreviations are used in this manuscript:

LCOS	Low cardiac output syndrome
ECMO	Extracorporeal membrane oxygenation
PICU	Pediatric Intensive Care Unit
CPB	Cardiopulmonary bypass
RACHS-1	Risk Adjustment for Congenital Heart Surgery Score
S_v_O_2_	Venous oxygen saturation
S_a_O_2_	Arterial oxygen saturation
D_av_SO_2_	Difference between arterial and central venous oxygen saturation
P_v_CO_2_	Venous partial pressure of carbon dioxide
P_a_CO_2_	Arterial partial pressure of carbon dioxide
D_av_CO_2_	Difference between arterial and venous partial pressure of carbon dioxide
VIS	Vasoactive–inotropic score
RRT	Renal replacement therapy
CABG	Coronary artery bypass graft
LVEF	Left ventricular ejection fraction
IABP	Intra-aortic balloon pump
ICU	Intensive care unit
RCT	Randomized controlled trial
